# Timing and Intensity of Light Correlate with Body Weight in Adults

**DOI:** 10.1371/journal.pone.0092251

**Published:** 2014-04-02

**Authors:** Kathryn J. Reid, Giovanni Santostasi, Kelly G. Baron, John Wilson, Joseph Kang, Phyllis C. Zee

**Affiliations:** 1 Department of Neurology, Northwestern University, Feinberg School of Medicine, Chicago, Illinois, United States of America; 2 Department of Preventive Medicine, Northwestern University, Feinberg School of Medicine, Chicago, Illinois, United States of America; Simon Fraser University, Canada

## Abstract

Light exposure can influence sleep and circadian timing, both of which have been shown to influence weight regulation. The goal of this study was to evaluate the relationship between ambient light, sleep and body mass index. Participants included 54 individuals (26 males, mean age 30.6, SD = 11.7 years). Light levels, sleep midpoint and duration were measured with wrist actigraphy (Actiwatch-L) for 7 days. BMI was derived from self-reported height and weight. Caloric intake was determined from 7 days of food logs. For each participant, light and activity data were output in 2 minute epochs, smoothed using a 5 point (10 minute) moving average and then aggregated over 24 hours. The mean light timing above 500 lux (MLiT^500^) was defined as the average clock time of all aggregated data points above 500 lux. MLiT^500^ was positively correlated with BMI (r = 0.51, p<0.001), and midpoint of sleep (r = 0.47, p<0.01). In a multivariable linear regression model including MLiT^500^ and midpoint of sleep, MLiT^500^ was a significant predictor of BMI (B = 1.26 SE = 0.34, β = 0.53 *p* = 0.001, *r* ^2^_Δ_ = 0.22). Adjusting for covariates, MLiT^500^ remained an independent predictor of BMI (B = 1.28 SE = 0.36, *β* = 0.54, p = 0.002, *r* ^2^_Δ_ = 0.20). The full model accounted for 34.7% of the variance in BMI (p = 0.01). Exposure to moderate levels of light at biologically appropriate times can influence weight, independent of sleep timing and duration.

## Introduction

Increased exposure to light late in the day and less exposure to bright light in the morning is often a consequence of sleep curtailment, and in particular with late sleep-wake timing [1], [2]. Several studies now indicate that morning light exposure influences body fat [3], [4] as well as the level of appetite regulating hormones [5]. However no published studies have investigated the influence of both light levels and sleep on body weight in humans.

Recent studies suggest that manipulating sleep duration and/or light exposure in humans results in alterations in metabolic function [5], appetite [3], and body fat [3], [4]. Light exposure of different wavelengths (i.e., 633 nm, 532 nm, 475 nm) in the morning for two hours immediately upon waking in sleep restricted (5 hours/night) individuals altered the levels of the satiety hormones, leptin and ghrelin [5]. Further support for the role of light in weight regulation comes from two intervention studies in obese women. In a study by Danilenko and colleagues, exposure to at least 45 minutes of morning light (between 6–9 am at 1300 lux) for 3 weeks in obese women resulted in reduced body fat and appetite that was not related to differences in photoperiod [3]. Similar findings were reported in a study by Dunai and colleagues that combined both light and exercise compared to exercise alone in obese women and found both groups had a significant difference in BMI but there were greater reductions in body fat in the women in the light and exercise group [4].

Evidence from animal studies also indicate that alterations in the duration of light exposure and the timing of feeding in relation to light exposure, can impair glucose metabolism and result in weight gain [6], [7], [8]. An important finding in these studies was that the increased weight gain was not associated with changes in caloric intake. Arble and colleagues [7] observed a greater weight gain in mice fed only during the light phase (rest period) compared to mice fed only during the dark phase (active period). This finding was further supported by Fonken and colleagues [8] who found that when mice were kept in constant light, they gained more weight than mice under a light/dark cycle. However, this effect was no longer observed when feeding was restricted to the clock time corresponding to the dark phase. Taken together, data from both animals and human studies suggest that light exposure may modulate metabolism and body weight/composition.

Short sleep duration and later sleep timing have been linked to higher BMI in multiple studies [9], [10], [11], [12], [13], [14]. These conditions increase the potential for exposure to light at inappropriate biological times (i.e. light at night and/or reduced morning light). Given the growing evidence for a role of light in regulating body weight, the goal of this study was to evaluate the relationship between the timing and duration of daily habitual ambient light exposure, sleep timing and duration with BMI. We hypothesize that the timing and intensity of light exposure (particularly in the morning) will be associated with a lower BMI independent of sleep duration and timing.

## Materials and Methods

### Ethics Statement

All participants provided written informed consent to participate in the study. This study was approved by the Northwestern University Institutional Review Board.

### Participants

Participants were adults recruited from the community through advertisements for a study of circadian rhythms and sleep patterns. The inclusion criteria from the initial telephone/email screening were age >18 years and no major unstable health conditions. Participants who were consented and completed wrist actigraphy and food diaries were included in this analysis. Participants with elevated depressive symptoms, as indicated by a score >20 on the Center for Epidemiologic Studies Depression Scale (CESD) [15] were excluded from these analyses. None of the participants reported employment involving shift work.

### Procedure

Participants underwent preliminary telephone or email screening to determine eligibility and willingness to participate in the study. Once informed consent was obtained, participants were provided with 7 days of diet logs, sleep logs, and a wrist actigraph (AW-L Actiwatch, Mini Mitter Co. Inc., Bend, OR) which was worn on the non-dominant wrist for at least 7 days. Participants were instructed to wear the Actiwatch on the outside of clothing at all times. In the daily diet logs the participants were asked to list a description of each food (quantity, preparation, name brand etc), the time and location of the meal or snack. In the sleep logs participants were asked to report sleep and wake timing, in combination with the actigraphy (Actiware-Sleep 5 software, Philips/Respironics) sleep and wake timing and sleep duration were determined.

### Measures

Participants were screened for depression with CES-D. Body Mass Index (BMI) was calculated as kg/m^2^ based upon self-reported height and weight. Season was determined by the time of year that the wrist actigraph was worn, Winter (December-February), Spring (March-May), Summer (June –August) and Fall (September –November), there was fairly even distribution of data collection during all four seasons.

### Dietary Assessment

Dietary intake was assessed using a diet log in which participants recorded all food and drinks for a 7 day period. We asked participants to record the time the food or drink was consumed, meal (breakfast, lunch, dinner, or snack), type of food with brand name if possible, the location of the meal or snack (i.e. home or restaurant), portion size, and whether it was a day they consumed less than a typical diet, more than a typical diet, or a typical diet. Along with their diet logs, participants were provided with two pages of instructions for completing diet logs. Instructions asked participants to include portion size (cups, ounces, and pieces), brand, information on preparation method (e.g. boiled, fried in oil, eaten with refuse), condiments and breaking foods into component parts (e.g. sandwich is two pieces of wheat bread, 2 oz of turkey breast). The second sheet was a portion size guide, and provided suggestions for how to judge portions without measuring (e.g. the size of a deck of cards, ping pong ball, your fist).

Diet logs were analyzed using publicly available nutrition information (www.sparkpeople.com) as well as restaurant and manufacturer websites. Caloric intake was computed for each day then the mean was computed for the 7 day period. Logs were considered valid if there were at least 2 weekdays and 1 weekend days completed. Dietary logs were excluded if total calories per day were <500 (this was the case for one participant). If participants had fewer than 7 days recorded, all of the available data was used; alternatively, if an excess of 7 days were completed, the investigators used the first 7 consecutive days that best coincided with actigraphy recordings.

### Sleep Timing and Duration

Sleep timing and duration were assessed using sleep logs and wrist actigraphy [16], [17]. The following variables were determined: sleep start, sleep end, and sleep duration. Rest intervals (inclusive of bedtime and waketime) were set by the investigators using the sleep logs as a guide [18], [19]. Sleep variables were calculated by the Actiware 5 software (Philips/Respironics) using default settings. Sleep start was defined as the first epoch, after the start of the rest interval, of the first consecutive 10 minute period in which all but one epoch was scored as immobile. Sleep end was defined as the last epoch, prior to the end of the rest interval, of the last consecutive 10 minute period in which all but one epoch was scored as immobile. Immobile is defined by the software when the number of activity counts recorded in that epoch is less than the epoch length in 15-second intervals. For example, there are four 15-second intervals for a 1-minute epoch length; hence, the activity value in an epoch must be less than four, to be scored as immobile. Wake threshold, which is the number of activity counts used to define wake, was set at medium (40 counts). Sleep duration was defined as the amount of time between sleep start and sleep end that was scored as sleep (an epoch is scored as sleep if the total activity counts ≤ wake threshold value). We calculated midpoint of sleep based on the average of the sleep onset and sleep offset for the 7 day period.

### Light Levels and Timing

**Figure 1:**
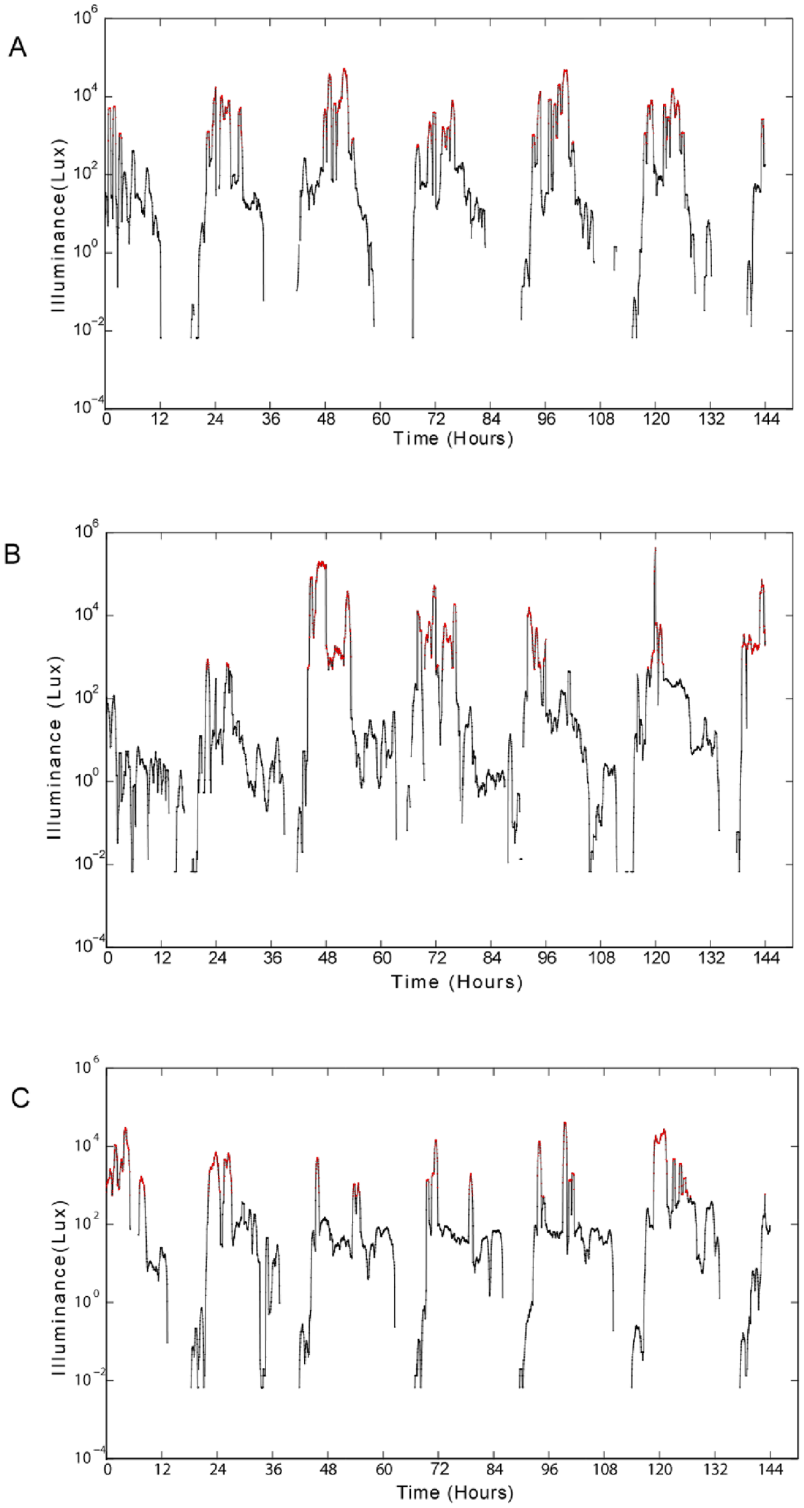
Representative log linear light plots of smoothed data across 7 days from three individual participants. Light data (lux) for up to 6.5 days is plotted by hour starting at 12 pm on day 1 (hours). The red data points and line indicate when the light level was greater than 500 lux. The data points with a zero lux value are not shown since the log of 0 is not a number.

**Figure 2:**
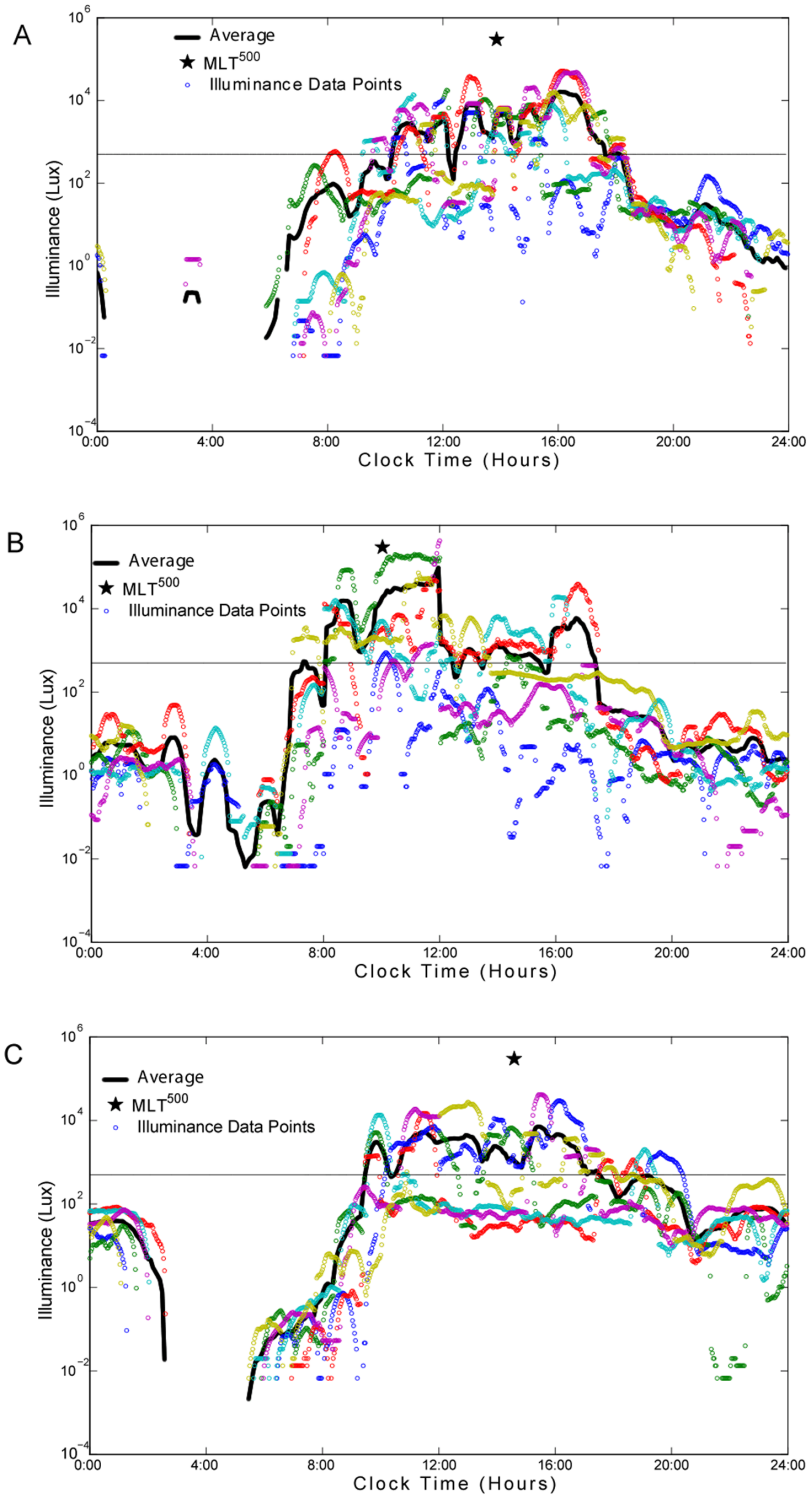
Representative log linear light plots from three individual participants. Individual light data (lux) for up to 7 days is plotted by time of day (clock time in hours). The smoothed average is shown as a bold line while the MLiT^500^ is shown as a star, the open circles represent individual illuminance data points and each day is represented as a different color. The 500 lux threshold is shown as a continuous horizontal line. Panels A–C represents light profiles with a range of MLiT^500^. Areas where there are no data points (night time in Panel A and C) are where the light level readings were zero since the log of 0 is not a number. Panel A, represents an individual with a MLiT^500^ at 13∶52 hrs; panel B represents an individual with an MLiT^500^ at 10∶02 hrs and panel C represents an individual with an MLiT^500^ at 14∶35 hrs.

Light levels were determined at the wrist using the AW-L Actiwatch (Mini Mitter Co. Inc., Bend, OR) [20]. Data were cleaned in Actiware 5 (Philips/Respironics), this involved excluding periods where the actigraph was taken off the wrist [21]. In order for a day to be considered valid and therefore included in the analysis it could not have more than four hours of excluded data in a 24 hour period. For each participant light and activity data were exported from the Actiware 5 program at a time resolution of 2 minutes (epoch). These exported data were first smoothed using a 5 point (10 minute) moving average (Figure 1) and then aggregated over 24 hours for each participant (Figure 2).

The following variables were calculated from this smoothed and aggregated data for each participant, time above threshold (TAT), mean light timing above threshold (MLiT) and standard deviation of the MLiT. TAT and MLiT were also calculated separately for the day-time (6 am–8 pm) and the night-time (8 pm–6 am). TAT is defined as the number of 2 minute epochs above a given threshold multiplied by 2 minutes. Mean light timing (MLiT) above threshold integrates information on the intensity (lux threshold), duration (number of 2 minute epochs above the threshold) and timing (clock time of each 2 minute epoch above the threshold) of light exposure. Individual level MLiT^C^ is formulated with general threshold C of LUX as:
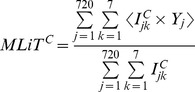
Where 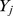 is the j^th^ epoch and 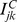 is 1 if LUX>C on the k^th^ day with indicators: j = 1,…,720; k = 1,…,7; and C = 500, 1000, 1500. Here j reaches 720 because the light exposure in LUX is measured every two minutes for 24 hours (720 = 24×60÷2). Thus, for example, MLiT^500^ of 720 minutes indicates that one’s light exposure being greater than 500 lux is on average centered around 720 minutes (or around 12 PM if the period starts at 12 AM) throughout 24 hours for the 7 days. MLiT^500^ was only available in 51 participants due to threshold requirements. Representative examples of individual profiles of light and the timing of MLiT^500^ are provided in Figures 1 and 2. The standard deviation of MLiT is defined as the standard deviation of all the times of the 2 minutes bins above a given threshold, and was determined to quantify the spread of the clock times above a given threshold.

### Statistical Analysis

Data were analyzed using SPSS v. 21.0 using bivariate correlations and multivariable linear regression analyses. In bivariate correlations, we tested correlations of BMI with sleep timing, duration, caloric intake and light variables, including TAT and MLiT at 100, 500, and 1000 lux for 24 hours. For models in which light was a significant predictor of BMI, the light variable was entered into the model to predict BMI controlling for midpoint of sleep. In the second model, we also controlled for relevant covariates including age, gender, season, activity counts (24 hours) [22], sleep midpoint and total sleep time by entering them as covariates in a regression equation. We also conducted sensitivity analyses on TAT and MLiT to evaluate thresholds ranging from 1–1400 lux for the following time periods 1) the entire day (24 hours), 2) day-time (6 am–8 pm), 3) night-time (8 pm–6 am) and 4) morning (8 am–12 pm). The times for day and night were selected to approximate times when there may have been natural light year round, other time windows were initially examined without significant changes to the results (for example day-time 9∶00 am–11∶59 pm and night-time 12∶00 am–8∶59 am to approximate the average sleep-wake schedule). In addition, a sensitivity analysis was conducted to evaluate the influence of measurement error on correlations with BMI. Statistical significance was defined as p<.05 on two tailed tests. Data are available upon email request to the senior author (Phyllis C. Zee).

## Results

Participant demographic, sleep, caloric intake and light characteristics are listed in Table 1. Average age of the participants was 30.6 years (*SD* = 11.7) and half of the participants were female. Average BMI was in the normal range (*M* = 24.0, *SD* = 4.2); 58% reported a BMI equal to or below 24. The average number of valid actigraphy days available was 6.2 (range 5–9 days). Average sleep start time was 01∶26 (02∶03) and average sleep end time was 08∶49 AM (02∶14). Average midpoint of sleep was 05∶12 AM (02∶14), and sleep duration was 6.2 hours (0.9). Average MLiT^500^ was 13∶05 (01∶46), average time above a threshold of 500 lux was 1.4 (1.3) hours. The standard deviation of MLiT was greater at the 100 lux threshold (4.4 hours) than at the higher thresholds (1.4-1.2 hours).

**Table 1:** Participant demographic, sleep, caloric, and light characteristics.

Variable	N	Mean (Stdev) or N (%)
**Sample characteristics**		
Age (years)	54	30.6 (11.7)
Sex	54	26 M 28 F
BMI	52	24.0 (4.2)
Daily caloric intake	54	2015 (545)
White N (%)	54	40 (74)
Season N (%)	54	
Winter		13 (24)
Spring		14 (26)
Summer		11 (20)
Fall		16 (29)
**Actigraphy Variables**		
Sleep start (hh:mm)	54	01∶26 (2∶03)
Sleep end (hh:mm)	54	8∶49 (2∶14)
Sleep duration (minutes)	54	375 (54)
Sleep midpoint (hh:mm)	54	05∶12 (2∶14)
Activity level (arbitrary units)	54	323,582 (96,510)
**Light Variables**		
Number of days	54	6.2 (0.77, range 5–9)
Time above threshold (TAT) 100 lux (hours)	54	4.3 (2.5)
Time above threshold (TAT) 500 lux (hours)	54	1.4 (1.3)
Time above threshold (TAT) 1000 lux (hours)	54	1.0 (1.1)
Mean Light Timing (MLiT^100^) above 100 lux (hh:mm)	53	13∶56 (01∶20)
Mean Light Timing (MLiT^500^) above 500 lux (hh:mm)	51	13∶05 (1∶46)
Mean Light Timing (MLiT^1000^) above 1000 lux (hh:mm)	44	13∶26 (01∶17)
Standard Deviation MLiT^100^ (hours)	53	04.42 (2.5)
Standard Deviation MLiT^500^ (hours)	51	01.49 (1.3)
Standard Deviation MLiT^1000^ (hours)	44	01.24 (1.09)

### Correlations between Timing of Light Exposure, BMI, Sleep and Calories

**Figure 3:**
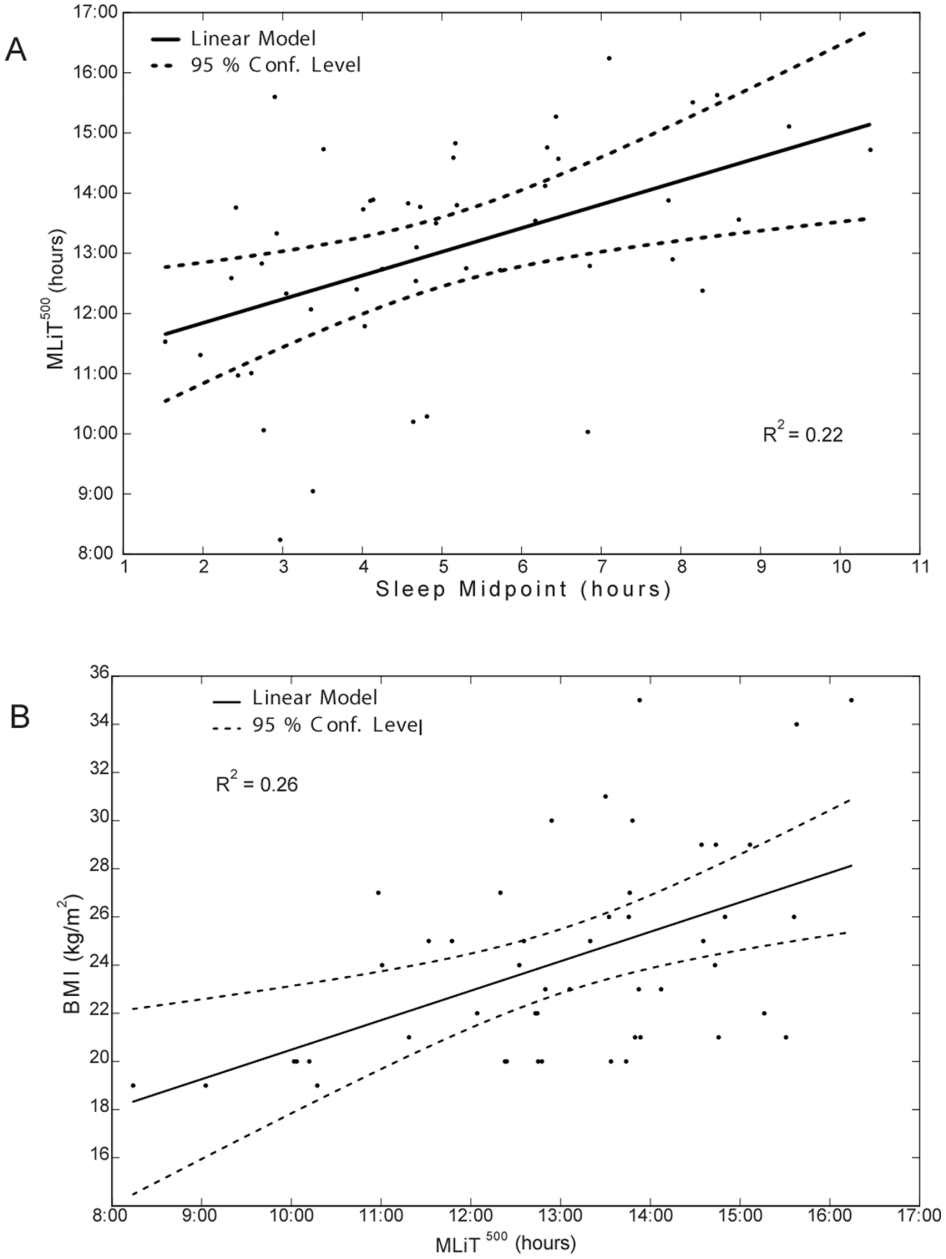
Association between MLiT^500^ and sleep midpoint (A) and between MLiT^500^ and BMI (B). The 95% confidence curves are designated with dotted lines.

Correlations are listed in Table 2. Of the light variables we tested, only MLiT^500^ was positively correlated with BMI (*r* = 0.51, *p*<.001; Figure 3B). Later midpoint of sleep was associated with later light exposure (MLiT^100^ *r* = 0.65, *p*<.001, MLiT^500^, *r* = 0.47, *p*<.001 (Figure 3A), MLiT^1000^ *r* = 0.48, *p*<.01) but was not associated with BMI or caloric intake. Sleep duration was not associated with timing of light, BMI or total caloric intake. Caloric intake was not associated with BMI, light, sleep timing or duration.

**Table 2:** Associations between body mass index and time above threshold, mean light timing, caloric intake and sleep.

	BMI	TAT 100	TAT 500	TAT 1000	MLiT 100	MLiT 500	MLiT 1000	Caloric Intake	Sleep Duration	Sleep Midpoint
**BMI**	–	.06	.14	.16	.11	.51***	.27†	.20	−.12	.12
**TAT 100**		–	.73***	.69***	.01	.04	.06	.04	.05	−.31*
**TAT 500**			–	.98***	.10	.13	.15	−.06	.08	−.18
**TAT 1000**				–	.08	.14	.18	−.04	.07	−.16
**MLiT 100**					–	.56***	.67***	−.09	.10	.65***
**MLiT 500**						–	.70***	−.14	−.00	.47***
**MLiT 1000**							–	−.11	−.10	.48***
**Caloric Intake**								–	−.26†	−.15
**Sleep Duration**									–	−.00
**Sleep Midpoint**										–

***p<.001 **p = <0.01,

† p = 0.07.

TAT: Time above threshold.

MLiT: Mean light timing.

### Multivariable Analyses

**Figure 4:**
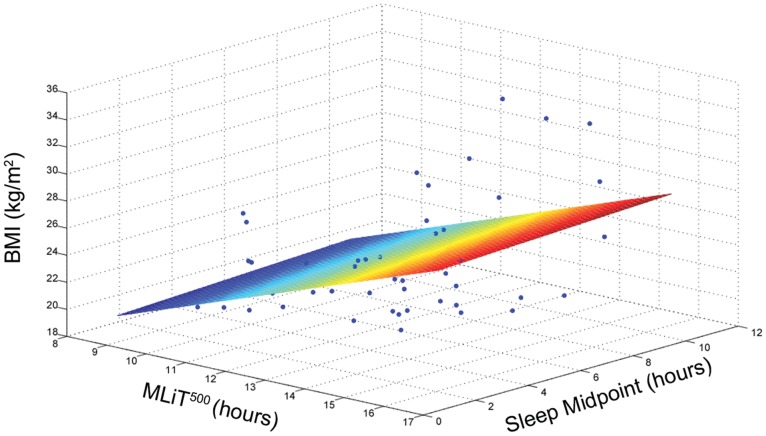
Multiinear association between BMI, sleep midpoint and MLiT^500^. This figure depicts a three dimensional relationship between BMI, MLIT^500^ and sleep midpoint.

In a multivariable linear regression model including MLiT^500^, sleep midpoint and BMI, MLiT^500^ remained a significant predictor of BMI (B = 1.26 SE = 0.34, β = 0.53 *p* = 0.001, *r* ^2^_Δ_ = 0.22, Figure 4). In the fully adjusted model, which adjusted for covariates, including age, gender, season, activity counts, sleep duration and sleep midpoint, MLiT^500^ remained a significant predictor of BMI (B = 1.28 SE = 0.36, *β* = 0.54, *p* = 0.001, *r* ^2^_Δ_ = 0.20). The full model accounted for 34.7% of the variance in BMI (*p* = 0.01).

### Sensitivity Analyses

Several sensitivity analyses were conducted aimed to assess the range of light level thresholds and times of day that were associated with BMI. Sensitivity analysis was conducted for four different windows of time: 1) the entire day (24 hours), 2) daytime (6 am–8 pm), 3) night time (8 pm–6 am), and 4) morning (8 am–12 pm). We also conducted sensitivity analyses to test measurement error in BMI.

### Sensitivity Analysis 24 Hour Day

**Figure 5:**
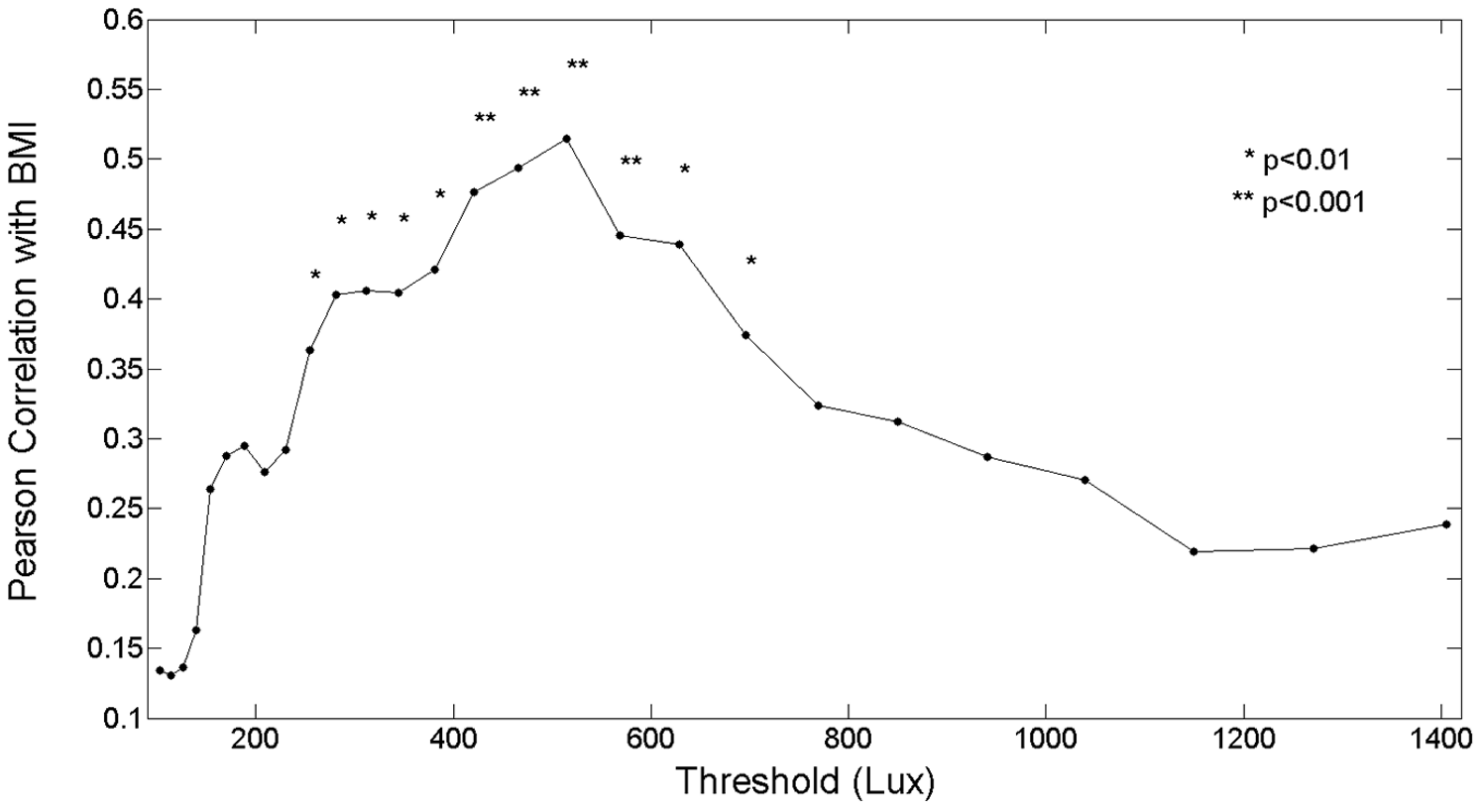
Pearson correlations with BMI by MLiT at various thresholds in lux. A range of values between 170 and 850 Lux demonstrates a statistically significant correlation with the strongest correlation at approximately 500 Lux.

A sensitivity analysis was conducted for time above threshold (TAT) and there were no associations between TAT at any light threshold and BMI. Figure 5 depicts sensitivity analyses testing associations between BMI and MLiT of light at different thresholds ranging from 100 to 1400 lux. This analysis indicated that MLiT^500^ had the strongest associations with BMI but there were statistically significant correlations between 170 and 850 lux. We also assessed our novel measure of mean light exposure time (MLiT) differently with a weight that incorporates light intensity. This weight is defined as a normalized (log transformed) light intensity, which serves as the distribution of light intensity during a 24-hour day and above a certain lux threshold, for example, 500 lux. Multiplied by this light intensity weight, the MLiT is expected to be closer to the period of major light exposure. Our assessment of the MLiT with this weight was quite consistent with the unweighted MLiT because the interval of the major light exposure occurred during the day (6 AM–8 PM). Due to the consistent occurrence of major light exposure during the day, the MLiT was robust either with or without the light intensity weight.

### Sensitivity Analysis for Day, Night and Morning

A sensitivity analysis was also conducted for light exposure only during the night-time (8 pm–6 am) and only during the day-time (6 am–8 pm) because different thresholds may be relevant at these time periods. There were no associations between TAT and BMI during the day-time or night-time. There was also no association between BMI and MLiT at night-time (1–100 lux) nor when only including the morning hours. The average MLiT^10^ at night-time was at 22∶48 (range 20∶57–01∶43). This lack of association between MLiT and BMI may be in part due to the very limited amount of TAT at higher light thresholds at night-time. The time above threshold at night-time for 10 lux was 2.6 (±1.6) hours, 50 lux was 34 (±36) minutes and for 100 lux was 14 (±22) minutes. During the day-time (6 am–8 pm) there was an association between BMI and MLiT at similar lux threshold levels as reported for the 24 hour day.

### Sensitivity Analysis for Self-reported BMI

In order to examine the influence of potential measurement error of the self-reported BMI, we conducted an analysis to determine the influence of random error in BMI on the strength of the correlation between BMI and MLiT. The following equation was used for this analysis: BMI_2_ = BMI_1_+0.1 •BMI_1_•ε (where ε is a random value from standard normal distribution and BMI_1_ is self-reported BMI and BMI_2_ reflects an error in reporting BMI_1_ of 10 percent). BMI_2_ was generated 1000 times. Even with adding 10% error, the average correlation between BMI and MLIT^500^ was r = 0.45 SD = 0.14 (95% CI 0.17, 0.73).

## Discussion

The results of this study demonstrate that the timing of even moderate intensity light exposure is independently associated with BMI. Specifically, having a majority of the average daily light exposure above 500 lux (MLiT^500^) earlier in the day was associated with a lower BMI. In practical terms, for every hour later of MLiT^500^ in the day, there was a 1.28 unit increase in BMI. The complete regression model (MLiT^500^, age, gender, season, activity level, sleep duration and sleep midpoint) accounted for 34.7% of the variance in BMI. Of the variables we explored, MLiT^500^ contributed the largest portion of the variance (20%).

Our results suggest that the relationship between light and BMI is not simply a function of the accumulated minutes of light during the day, but more importantly the temporal pattern of light exposure above a biological threshold. The threshold/intensity of light is also an important factor since the relationship between MLiT (24 hours) and BMI was only significant for light intensities above 170 lux and up to 850 lux in our sensitivity analyses. To put these light levels in perspective, normal room light is typically between 150–500 lux [23]. In this sample on average, only 4 hours per day was spent above 100 lux and 1 hour above 1000 lux suggesting that in general light exposure is similar to that of indoor lighting for much of the day. The time spent above 1000 lux in this sample was similar to that reported by Staples et al. 2009 [2] and slightly shorter than that reported by Goulet et al. 2007 [1]. Both biological and behavioral factors related to ambient light exposure patterns may play a role in explaining why these particular light levels are important for the association between light and BMI. Since most participants had light above 100 lux for at least a few hours each day, only light thresholds higher than this could discriminate across the range of BMI. Whereas the cut off at the higher light levels was most likely due to fewer individuals having ambient light exposure levels at or above 1000 lux.

It is also possible that the natural changes in the intensity and wavelength composition of light in the morning compared to the afternoon/evening [24] may in part explain our finding for a differential effect of earlier vs. a later daytime light exposure pattern and BMI. For example, there is generally a higher amount of blue light (shorter wavelength) in the morning. [25] Blue light has been shown to have the strongest effect on the circadian system, including the suppression of nocturnal melatonin secretion [26].

An interesting and perhaps unexpected result was the lack of correlation between BMI and TAT or MLiT at night-time at any threshold, including very low light levels of 10 lux or lower. One possible explanation for the lack of association in this study between light at night and BMI may be due to the very limited amount of even moderate levels of light at night in this sample. Thus, our results do not address the issue of the potential relationship of brighter light exposure during the night and BMI.

Although our data indicate that those with more light exposure above key thresholds earlier in the day are more likely to have a lower BMI, there was no association between MLiT and BMI for the morning hours (8 am–12 pm) only. This finding supports the hypothesis that the pattern of light exposure across the entire day is important for weight regulation. This is complicated by the close link between MLiT and sleep timing, and for some individuals, “morning” as defined by clock time may be quite different than their “biological morning”. In the studies that administered morning light and found reductions in body fat [3], [4], normalization of the timing of the sleep-wake cycle relative to light exposure, may have played a role in the effect. Since in these studies the timing of light exposure was always at a specific clock time (e.g. 45 minutes of exposure between 6–9 am), participants needed to be awake, to be compliant with treatment.

In our data, sleep timing was associated with the timing of light exposure (24 hours) at all the thresholds examined (MLiT^100–1000^), such that that a later sleep midpoint was associated with a later MLiT. However, unlike previous reports, the timing of sleep was not directly correlated with BMI in this study. This lack of association between sleep measures and BMI may be because of the larger range of sleep times. In this sample participants with earlier sleep and wake times (early chronotype) were included, whereas, in our previous report [10] only intermediate and evening (late) types were included. Unfortunately we are underpowered (N = 8) to investigate whether BMI is associated with sleep timing in morning chronotypes alone. There was also no association in this study between sleep duration and BMI, nor between sleep duration and light exposure. This may be due to a difference in the way that sleep data from different studies has been collected (subjective vs objective) and analyzed, since the association between sleep duration and BMI has typically been reported when self-reported sleep duration was treated as categorical variable [27]. Another factor could be that the median BMI is this sample is in the normal range (23 kg/m^2^). While there was no association between sleep timing and duration with BMI in this sample, the relationship between sleep, light exposure and body weight is likely to still be important. There is evidence from a recent experimental study for an interaction between sleep duration and light exposure with metabolic hormones. Sleep restricted to 5 hours per night paired with light exposure in the morning resulted in an increase in leptin and a decrease in ghrelin compared to the dim light condition [5].

Our findings, similar to those from two different animal models [7], [8] found that changes in the timing of light exposure were associated with body weight independent of caloric intake. One possible mechanism linking light directly to BMI, rather than caloric intake may be the influence of light on the expression and secretion of hormones, such as melatonin. In addition to its circadian timing effects, light exposure history during the day can alter nocturnal levels of melatonin [28] and sensitivity of the circadian clock to light [29], [30], [31]. These effects of light may play a role in metabolism and weight regulation. It has been shown, for example, that in middle-aged rats daily nocturnal melatonin administration for 3 weeks reduced weight gain in response to a high-fat diet and decreased nighttime plasma leptin concentrations, independent of total food consumption [32]. Alteration in melatonin level has also been shown to affect insulin sensitivity [33], [34], and recent studies in humans suggest that a low melatonin level is a risk factor for type 2 diabetes [35]. Future studies are needed to determine whether the influence of light on BMI is mediated by its effects on melatonin and/or circadian timing and amplitude [34], [35], [36]. Other potential mechanisms include the impact of light on sleep quality and autonomic function, [37], [38], [39] which can directly or indirectly affect metabolism and energy balance. For example, light exposure in the blue range (460 nm) in the evening may alter the dynamics of slow wave and rapid eye movement sleep [40], and such changes in sleep have been shown to affect metabolic function [41].

The limitations of this study include lack of random selection from a nationally representative sample and use of self-reported diet and BMI which may have resulted in measurement error [42]. After accounting for a 10% variation in BMI, the association between MLiT and BMI remained significant in this study. In addition, since young and normal weight participants (such as in this study) typically demonstrate a higher correlation between self-reported and objective BMI [43], one would expect a lower level of reporting bias. Ambient light levels were measured at the wrist and thus may not be representative of light intensity reaching the eyes, limiting our ability to determine the absolute light values for the biological effect of light on BMI. However, a study comparing light levels measured near the eye, to levels at the wrist, indicates that at light levels less that 5000 lux the readings from each device were fairly similar [44]. Another potential concern is the effect of clothing obscuring the light sensor. To limit this all participants were instructed to wear the device on the outside of clothing. In addition, controlling for season, which may impact the likelihood of the device being covered by clothing, did not significantly impact the relationship between MLiT and BMI. Even with these potential limitations, the ambient light values measured at the wrist do represent relative changes in light levels between individuals and within an individual across the day, and therefore do not limit the assessment of the timing of light exposure. The cross sectional study design does not allow us to directly infer directionality of the relationship between light and body weight.

In conclusion, the findings of this study indicate that the temporal pattern of light exposure during the daytime can influence body weight independent of sleep timing and duration. Further studies are needed to understand the causal relationship and mechanisms linking biologically appropriate and inappropriate light timing with weight. Nevertheless, light is a powerful biological signal and appropriate timing, intensity and duration of exposure may represent a potentially modifiable risk factor for the prevention and management of obesity in modern societies.
